# COVID-19: additive manufacturing response in the UK

**DOI:** 10.2217/3dp-2020-0013

**Published:** 2020-12-07

**Authors:** Elen J Parry, Craig E Banks

**Affiliations:** ^1^Faculty of Science and Engineering, Manchester Metropolitan University, Chester Street, Manchester, M1 5GD, UK

**Keywords:** additive manufacturing, agile manufacturing, COVID-19, healthcare, medical devices, medical equipment, personal protective equipment, regulatory approval

## Abstract

Severe acute respiratory syndrome coronavirus 2, a novel coronavirus, caused global disruption specifically in linear supply chains. Increased demand for already disrupted services led to a global shortage of medical equipment and personal protective equipment. Use of additive manufacturing (AM) processes by the manufacturing community has shown great innovation, agility and flexibility to fill supply chain gaps and meet shortfalls. In the context of contingency reaction to a global healthcare emergency, decisions have had to be made quickly, in some cases bypassing device safety regulations. This concentrated and spontaneous use of AM has highlighted the challenges and risks of such innovation, which we discuss in relation to the UK’s current regulatory landscape. We have discussed lessons learned and the potential future impact upon wider use of AM in healthcare.

In December 2019, Wuhan, China, became the epicentre of a fast-spreading novel virus [1]. On 30 January 2020, the outbreak was declared a public health emergency and by 11 March 2020 the severe acute respiratory syndrome coronavirus 2 was categorized as a pandemic [2]. The severe acute respiratory syndrome coronavirus 2, herein discussed as COVID-19, pandemic has brought unprecedented challenges, one of which being the critical shortage of supplies including medical equipment (ME) and personal protective equipment (PPE).

The resilience of supply chains (SC) has been tested with the closure of crucial facilities and drastically heightened demand. The transition from linear SC operating in silos, to intertwined digital supply networks is underway [3] and is now more necessary than ever. Enabled by the Internet-of-Things, supply networks are dynamic and interconnected, proving increased resilience to disruption. Improved visibility and real-time mapping of supply and demand is emerging [4], which could prove essential in meeting urgent demand for critical supplies.

In an attempt to meet demand, the WHO called for governments to increase manufacturing by 40% [5], which resulted in the global manufacturing industry supporting the response to COVID-19 by producing designs, ME, medical testing equipment, PPE and manufacturing equipment. Many firms who volunteered their support to the government in the UK (GOV-UK) were provided with specification documents for ventilators to be used in hospitals by the UK Medicines and Healthcare products Regulatory Agency [6]. Automotive and fashion companies are some of the many named working in conjunction with GOV-UK to assist with design and production efforts [7].

## The role of additive manufacturing during a global crisis

### Manufacturing flexibility

Extreme shortages in PPE and ME meant a new approach to meet demand was vital. Additive manufacturing (AM) is a term for a group of fabrication techniques which builds parts by adding material layer-by-layer until a part is fully formed. The additive nature of the process allows for the fabrication of complex geometry, which unlike traditional subtractive manufacturing methods, do not require specialist tooling [8]. Hence, production can switch from one part to another almost instantly, which has been demonstrated by those with AM capabilities producing products on demand.

### Increased accessibility

More recent democratization of AM has seen the technology increasingly present in universities, schools, makerspaces and for many enthusiasts, in their homes. Together, this widespread community of AM users formed a response network to contribute to the PPE and ME relief effort, by manufacturing devices on their 3D printers. These often collaborative efforts are being referred to as a ‘citizen supply chain’. The AM community have actively shared designs, digital files and knowledge through digital networks making it easy for anyone with access to a 3D printer to contribute. The citizen supply chain has been supported by larger companies in the AM industry, including Prusa^®^ (Prague, Czech Republic) and Copper 3D^®^ (Santiago, Chile). A widely discussed Prusa face shield design was published on 18 March 2020 and as of 30 April 30 2020, it had been downloaded approximately 200,000-times [9], with community users being encouraged to improve the design [10]. Similarly, Copper 3D encouraged community collaboration to improve the design of their ‘Nanohack’ mask, which is an open-source antimicrobial face mask [11].

Social media (SM), coupled with increased accessibility to AM facilities, is believed to be a key driver of the overwhelming response from the AM community. A study reviewed the impact of SM during the COVID-19 AM response between 1 January 2020 and 14 April 2020 through utilizing SM listing software to explore keywords relating to AM, PPE and ME. The study presented an approximate total reach of 7.2 billion SM responses with more than 18,000 individuals from 36 countries contributing to the AM effort for COVID-19 [12].

### Localized manufacture

With such wide contribution, the citizen supply chain is naturally decentralized, and is operating across the globe through digital connection. Decentralized manufacturing generally offers some protection against external disruption and the delays seen in centralized manufacturing models [13] by utilizing shorter and more direct SC, in turn allowing for faster acquisition of essential products and components [14,15]. By operating locally, manufacturers can improve their responsiveness and directly supply demand by reducing supply chain complexities [16]. Positive outcomes of localized manufacture include reduced lead times and in some cases lower costs, however concerns have been raised around reputation and regulation [17].

The COVID-19 response effort saw individuals and organizations supplying their local hospitals, care homes, shops and schools with PPE when it could not be sourced through standard procurement routes. This model continued by many until conventional manufacturing processes recovered from disruption and were able to catch up and meet demand.

### Reduced time-to-market

**Figure 1.**
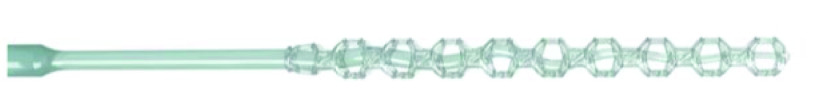
Resolution Medical lattice swab. Figure taken with permission from Resolution Medical, 2020.

**Figure 2.**
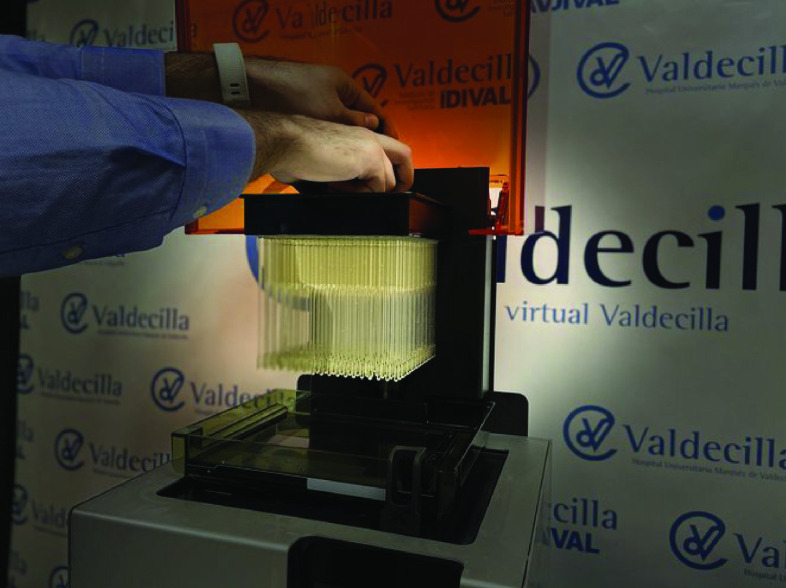
Test swabs manufactured on a Formlabs 3D printer. Figure taken with permission from Formlabs, 2020.

As well as many changing their production lines to meet current needs, additional benefits of AM such as reducing time-to-market have been demonstrated. Companies have worked to quickly release certified medical devices (MD) and ME. Examples include Resolution Medical^®^ (MN, USA), who developed US FDA approved lattice swabs using Carbon™ AM technology [18] shown in Figure 1. A similar collaborative response with Concordance Healthcare Solutions^®^ (OH, USA) has led to the production of approved swabs, shown in Figure 2, using Formlabs Stereolithography AM technology [19], which is capable of producing 650 swabs on a single 3D printer in 24 h [20].

## Risks & challenges

### Product safety

Despite discussed benefits, use of AM within nontraditional routes raises a number of concerns and questions around safety. Established MD manufacturers, such as Resolution Medical and Concordance Healthcare Solutions, obtained regulatory approval through their already established regulatory pathways. For other inexperienced manufacturers, or those from citizen SCs, the safety of the devices produced is largely unknown. Determining product safety however, is not as straight forward as it might seem, and is arguably an unrealistic expectation for many under these unprecedented circumstances.

AM as a process brings its own set of complexities. There are numerous types of AM technologies, each with frequently expanding material libraries [21], meaning different material and process configurations naturally result in different part properties, and therefore, performance. Fused filament fabrication (FFF) is believed to be the most used type of AM by the citizen supply chain in responding to COVID-19 equipment shortages due to the wide availability of low-cost desktop 3D printers which are relatively simplistic and easy to use [22]. FFF in particular produces anisotropic parts due to the layering nature of the technology [23]. This means parts are weaker in the direction they were built and quality is often considered lower than alternative AM methods [8]. This can be problematic for functional devices, as delamination of layers can cause parts to break easily, which directly influences the safety of devices manufactured using FFF.

Despite the above limitation, parts produced using this technology can still be highly functional and effective when the process limitations are managed accordingly, and parts are appropriately designed for the AM process. Variations of this scenario are applicable across all AM technologies, and in fact all manufacturing methods in general. Therefore, knowing how to design for different manufacturing processes is an important but often overlooked skill, especially within AM, which could easily result in device failures [24]. To optimize part performance, the designer requires an understanding of the intended manufacturing technology, as well as the operation and control measures required during the process, to achieve optimal performance and quality [25].

Appropriate material selection is another important factor influencing the safety and suitability of AM parts. For example, devices intended to be in contact with the human body, such as facemasks must be biocompatible, and safe for skin contact. Biocompatibility requirements are application specific and depend on factors such as the type and duration of contact [26]. Biocompatible safety is not assured by appropriate material selection alone. AM processes can alter material properties and performance [27,28] and in some cases, particularly relating to post-production methods, expose parts to additional chemicals or materials which may alter part safety and performance [29]. Risks of toxicity are considerably lower for devices used outside of the body as opposed to other types of high-risk devices and equipment, however, parts could still pose a risk to the user if not managed correctly.

Sterilization is a common safety requirement in healthcare, and is particularly relevant to COVID-19 to ensure externally sourced PPE and ME is not contaminated. A common sterilization process is autoclave which exposes parts to high temperatures of between 121 and 132° C while exposing them to pressure [30]. As the majority of 3D printed parts are made from thermoplastics with low melting temperatures, sterilization processes such as autoclave are likely to deform or degrade parts [30,31]. Deformation in particular could increase risk to the user by affecting the functionality of PPE or ME.

The potential risks outlined above are rarely problematic under normal circumstances. This is due to all ME and PPE being highly regulated and required by law to meet specific safety standards, which together, consider the product’s design, materials, manufacturing processes and quality control procedures to ensure a safe and effective product. Products conforming with all relevant health and safety standards are marked with a CE-mark by notified bodies, who are responsible for enforcing regulation in the UK and wider European Union (EU). This allows manufactures to put products on the market within the EU and provides users with confidence that equipment is safe and effective.

However, at a time of critically urgent demand in responding to the COVID-19 equipment shortages, regulated pathways were unable to meet demand, and simply unequipped to assess every device being made and used for conformity. This is particularly relevant to products born out of the citizen supply chain for a number of reasons. First, it is unrealistic for non-MD manufacturers to understand and comply with regulations. Compliance is complex and can be challenging for even well-established certified manufacturers, especially within the context of AM, which is a relatively new method of manufacturing in healthcare with its own set of challenges. Second, localized manufacture is often disconnected from traditional routes meaning devices are manufactured and distributed without passing through any official checkpoints, making identification and traceability almost impossible.

The responsibility of using these products therefore relies with the institutions which receive them. Use of unregulated PPE has been seen in healthcare institutes out of necessity where there was no available certified alternative. In these cases, risk assessments were conducted locally and specific standard operating procedures were created to mitigate risk [32,33]. AM was essential for many in managing this interim period until SCs resumed and conventional manufacturers were able to scale production to meet demand. The urgent and extensive use of AM has highlighted important considerations associated with widespread use of the technology in a medical context. It has also forced clarification of the regulatory landscape, which is thought of by many as being an unsuitable pathway for AM devices [34].

## Regulatory landscape for AM

Under COVID-19 circumstances, the route to compliance remains largely unaltered. It was made possible to apply for exceptions through contacting the Medicines and Healthcare products Regulatory Agency with a detailed description of the product and why it does not have a valid CE mark. The application must discuss why alternative products would not be appropriate for use and attach clinical justification for requesting exemption. Intent to obtain a CE mark must be demonstrated by disclosing the expected timescale before gaining or re-gaining CE verification [35]. Evidently, exemption is not an easy route to conformance and cannot be granted without extensive documentation, making it largely unsuitable for most individual or low volume COVID-19 related response outcomes.

Regarding PPE, COVID-19 regulated PPE being purchased by GOV-UK or the UK National Health Service was exempt from conformity assessment for a limited time, provided it met essential safety requirements. For any other PPE necessary in the context of COVID-19, it must meet the essential safety requirements and conformity assessment procedures must have been started via a notified body [7].

In response to the citizen supply chain, the British Standards Institution (BSI), the national standards body in the UK, released a guide specifically for organizations, schools and universities manufacturing PPE with no prior experience to support these initiatives. Although popular belief among the AM and wider communities is that ‘any PPE is better than no PPE’, the statement is specifically addressed as incorrect by BSI. Inefficient PPE is likely to prompt a false sense of security meaning wearers may take less care than usual under the assumption they are protected, therefore putting them at greater risk [36]. BSI confirm that it “*is the legal responsibility of anyone producing PPE to ensure it protects the healthcare worker and is fit for purpose”* [36].

Many contributors have unknowingly accepted responsibility for the ME and PPE they are producing whether it has been sold or donated free of charge. Similarly, many were unaware that it is a requirement for MD manufacturers in the UK to hold extensive quality management system. Documentation including a quality manual, documented procedures and records demonstrating effective planning, operation and control of processes are all quality management system requirements, which could be subject to unannounced audits to ensure regulatory compliance [37]. For each type of MD, a technical file must be available which outlines product specifications, including procedures for manufacturing, storage, packaging, handling, distribution, measuring and monitoring; instructions for installation, use, care or servicing and product labeling [38]. Evidently, compliance is an extensive process which requires full commitment from a manufacturer. Furthermore, compliance is particularly challenging in the AM field, especially for less-commonly explored technologies such as FFF, which are currently undergoing research and testing to fully understand the performance and capabilities of the technology which is not yet known. These challenges directly relate to quality and safety issues which must be resolved for more widespread adoption [39].

The expectation of the citizen supply chain to comply with ME and PPE regulations is unrealistic, especially due to the majority being unaware of the expectations. Steps to ensure safety are absolutely necessary to protect healthcare workers, patients and other product users, however, the generous response from noncertified manufacturers and the citizen supply chain was greatly appreciated by many.

## Lessons learned

COVID-19 has accelerated some of the questions and technical issues of using AM in healthcare while presenting new challenges, such as mass accessibility and an open pathway for non-CE marked devices and equipment to enter society. AM has shown to be a highly effective tool to rapidly design and produce clinically certified MDs and equipment on-demand, which previously could have taken between months and years to develop. Nonprofessional AM has also prompted remarkable innovation and collaboration from AM communities, with a significant number of low risk devices, PPE and ME being produced locally.

This increased use has highlighted some issues specific to the opensource nature of AM and digital manufacturing techniques. Intellectual property (IP) has been a concern throughout the rise of AM relating to the unauthorized reproduction of protected products and components [40]. The venturi valve, a component in respiratory equipment, has been central to discussions around IP and copyright infringement [41]. The valve, subject to copyright and patent covers, was reverse engineered and the digital file was made available online to facilitate local production of these essential components [42]. Emergencies resulting in life-or-death decisions may be justification for full use of a design regardless of IP [42]. Moreover, it would be unpractical for legal action to be taken due to the vast number of design variations and possible cases of infringement. However, the importance of understanding IP has been highlighted, and precautions must be taken to ensure the severity of infringement is understood, and is not regarded as acceptable when associated with AM generally.

Limitations around functionality and quality have been addressed, and in response officials clarified the enforcement of a no-CE mark, no use policy in healthcare and social care settings. The positive contributions AM has provided are undeniable, so instead of the ‘all-or-nothing’ approach set out by the BSI and UK authorities, a collaborative approach may be more effective for managing an AM response in similar future scenarios. Although it is highly unrealistic to assess the conformity of every device, steps could be taken to enforce a low-level of standardization by providing approved design files, cleared of IP restrictions, that meet the EU MD regulation. Approved digital manufacturing files, downloadable with specific manufacturing instructions and a simple set of quality check procedures could be a preferable approach. Although this by no means guarantees safety, it is likely to be more effective than multiple different ‘do-it-yourself’ attempts from inexperienced members of the AM community wanting to contribute however they can.

**Figure 3.**
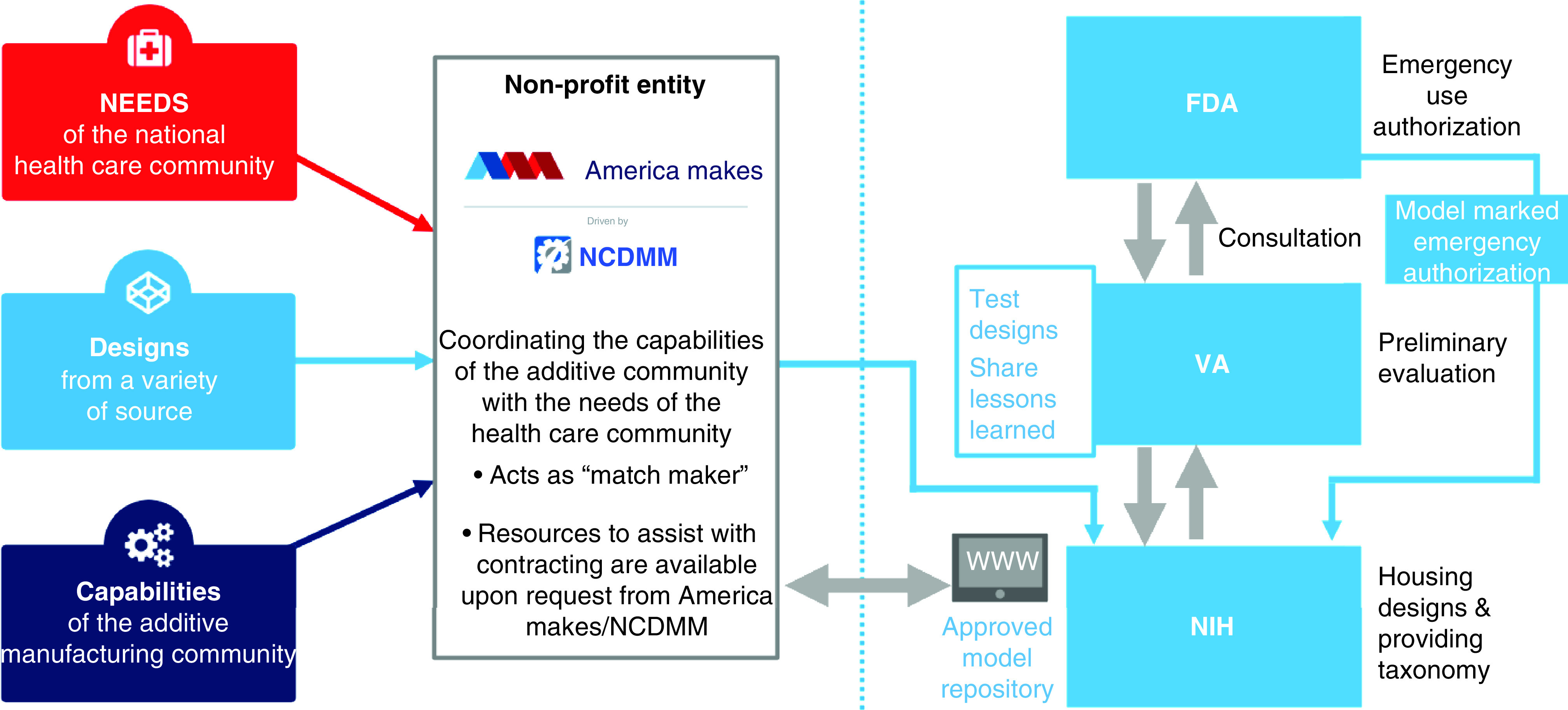
America Makes 3D printing response to COVID-19. Figure taken with permission from Fighting COVID-19 with 3D printing: America Makes Responds, 2020.

A similar initiative by America Makes, a non-profit AM innovation institute in the USA, was implemented. A collaboration between the FDA, Department of Veteran’s affairs and National Institutes of Health was set up, with America Makes as the central hub. Their efforts, visualized in Figure 3, collated the needs of healthcare workers, the designs created in response and the volunteered manufacturing capabilities of the community. This allowed for designs to be reviewed and progressed to a *clinical use review* where designs could be authorized for emergency use by the FDA, or either optimized for community use or returned with a warning of safety implications [43]. This kind of approach can be used to reduce risk, increase confidence and facilitate a more aligned and efficient response.

## Conclusion

In summary, the COVID-19 AM response has been overwhelming. The benefits of AM to rapidly design, innovate and produce critically urgent parts the most unprecedented challenges will be remembered. Contributions from distributed supply networks were seen globally, which in turn, highlighted intended use and product safety as the leading concern. Overcoming challenges relating to standardization and regulation across the entire AM industry could diminish some of the safety concerns identified. This unique situation has provided a concentration of AM use, teaching the industry valuable lessons, which will undoubtedly contribute toward progressing the field.

## Future perspective

The COVID-19 crisis has provided an opening for AM to demonstrate its potential where reduced lead-times are required and supply chain gaps are presented. A more realistic vision for AM has been demonstrated and the concerns relating to widespread AM use in healthcare have been highlighted. Through seeing the benefits, many companies are expected to reconsider adopting AM technologies as part of their day-to-day operations, not necessarily to replace their conventional methods but to support and enhance conventional methods. Wider use of digital integrated supply networks is expected, as well as heightened demand for clarified legislation and official guidance around AM adoption and good practice.

Increased attention toward the clinical certification of AM devices is likely to prompt further guidance for regulatory compliance, specifically for AM processes. Extended use of AM in healthcare requires more comprehensive safety standards and regulatory frameworks relating to use of AM technology, which will naturally progress from an increased use and understanding of AM for regulated products. The AM response to COVID-19 has highlighted the lack of standardization in the industry, whether that is through available guidance, hardware capabilities, quality of materials or standard operating procedures. A positive outcome for the AM industry would be the development of more guidance and a collation of standardized tests for AM parts. These outcomes could be hugely impactful in widening AM adoption in the healthcare industry.

In line with increased adoption however, the AM community as a whole have a responsibility to ensure the field grows in a conscious and sustainable manner. The AM response to COVID-19 could set a dangerous precedent for encouraged use of disposable plastic products. The question “Just because we can, does that mean we should?” is of utmost importance for the responsible growth of AM.

**Executive summary**

**Introduction**

- Severe acute respiratory syndrome coronavirus 2 is introduced and the critical shortage of medical equipment is identified.
- The progression to digital supply networks from linear supply chains is highlighted.
- The WHO called for a manufacturing increase to help meet demand.

**The role of additive manufacturing**

- Accessible additive manufacturing (AM) technology enabled a distributed network of makers were able to contribute to the relief effort forming a citizen supply chain.
- Social media and digital file sharing platforms drove collaboration.
- Localized manufacturing reduced supply chain complexities but raised concern around regulation.
- Medical equipment was quickly designed and manufactured using AM techniques.

**Risks & challenges**

- Product safety is a concern from inexperienced manufacturers.
- The challenges of regulatory approval are discussed.
- Limitations of AM technology are highlighted and their potential safety implications are discussed.
- The differing levels of complexity between AM technologies is discussed, along with the potential quality issues.
- Material challenges such as biocompatibility and sterilization are discussed in a safety context.
- The use of unregulated products is discussed and challenges of enforcing compliance for unconventional routes are highlighted.

**Regulatory landscape for AM**

- The traditional route to regulatory compliance, and the exceptions made for COVID-19 circumstances are discussed.
- The British Standards Institution introduced new guidance and resources for inexperienced manufacturers producing personal protective equipment but emphasize that anyone producing such equipment is legally responsible for it.
- The authors state the regulatory expectations for inexperienced manufacturers as unrealistic.

**Lessons learned**

- The challenges associated with intellectual property, copyright and AM are discussed.
- The authors suggest an alternative pathway for inexperienced manufacturers to contribute with manufacturing efforts by downloading approved files, instructional resources and quality check procedures.
- An initiative by America Makes is discussed as a positive solution which collaborates with official bodies to reduce the discussed risks.

**Future impact**

- Improved guidance for regulatory compliance through AM is expected.
- COVID-19 efforts have highlighted the lack of standardization in the AM industry, which is likely to result in improved standardization of software, hardware and test methods.
- Responsible and sustainable growth of the industry is necessary.
